# Supplementary material for: Identification of photoperiod-induced specific miRNAs in the adrenal glands of Sunite sheep (Ovis aries)
Source: Front Vet Sci. 2022 Jul 22;9:888207. doi: 10.3389/fvets.2022.888207 (PMC9354845; doi:10.3389/fvets.2022.888207)
Supplement: Supplementary file 1 [file Data_Sheet_1.ZIP › Supplementary file/miRDeep2 file.zip!/miRDeep2 file/Know_Structure/SP42A2/oar-mir-370.pdf]

miRBase precursor : oar-mir-370
Total read count : 14220
oar-miR-370-5p read count : 41
oar-miR-370-3p read count : 14165
remaining reads : 14

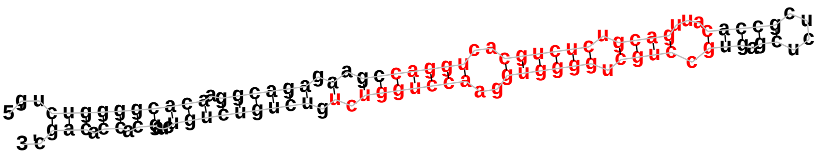

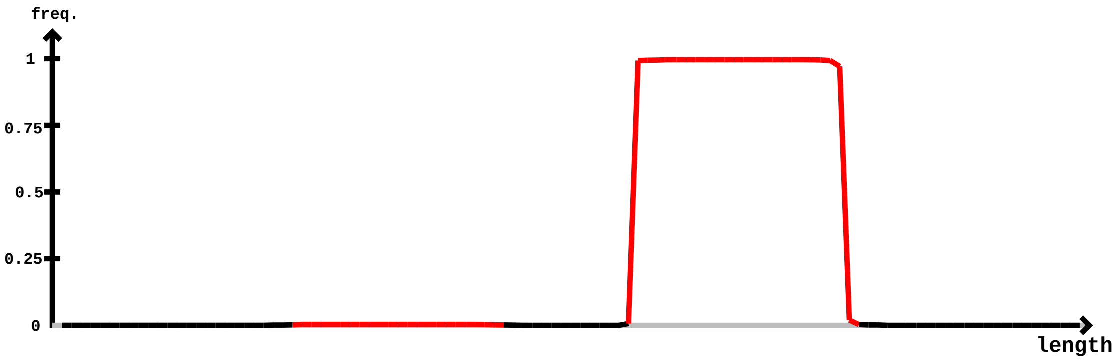


```
                                                                oar-miR-370-3p
                                oar-miR-370-5p
5'- gucuggggcacaaggcagagaagccaggucacgucucugcaguuacaccgcucucgagugccugcugggguggaaccuggucugucugucugucuagcaccacagc   -3'   exp
    ..((((((((((.((((((.(.(((((((..((((((.((((...(((((....)).))).)))).))))))..))))))).).)))))))))...)).)).))).    reads    mm    sample
    gucuggggcacaaggcagagaagc..................................................................................    1        0     seq
    ....ggggcacaaggcagagaagc..................................................................................    1        0     seq
    .....................agccaggucacgucucugcag................................................................    1        0     seq
    .....................agccaggucacgucucugcagu...............................................................    4        0     seq
    .....................agccaggucacgucucugcaguu..............................................................    7        0     seq
    .......................ccaggucacgucucugcag................................................................    1        0     seq
    .......................ccaggucacgucucugcagu...............................................................    1        0     seq
    .......................ccaggucacgucucugcaguu..............................................................    6        0     seq
    .......................ccaggucacgucucugcaguua.............................................................    4        0     seq
    ........................caggucacgucucugcagu...............................................................    1        0     seq
    ........................caggucacgucucugcaguu..............................................................    5        0     seq
    ........................caggucacgucucugcaguua.............................................................    3        0     seq
    ........................caggucacgucucugcaguuac............................................................    9        0     seq
    ........................caggucacgucucugcaguuaca...........................................................    10       0     seq
    ........................caggucacgucucugcaguuacac..........................................................    1        0     seq
    ..........................................................ugccugcugggguggaaccugg..........................    8        0     seq
    ..........................................................ugccugcugggguggaaccuggu.........................    87       0     seq
    ...........................................................gccugcugggguggaacc.............................    4        0     seq
    ...........................................................gccugcugggguggaaccu............................    11       0     seq
    ...........................................................gccugcugggguggaaccug...........................    28       0     seq
    ...........................................................gccugcugggguggaaccugg..........................    305      0     seq
    ...........................................................gccugcugggguggaaccuggu.........................    13424    0     seq
    ...........................................................gccugcugggguggaaccugguc........................    237      0     seq
    ...........................................................gccugcugggguggaaccuggucu.......................    15       0     seq
    ............................................................ccugcugggguggaaccugg..........................    1        0     seq
    ............................................................ccugcugggguggaaccuggu.........................    13       0     seq
    ............................................................ccugcugggguggaaccugguc........................    2        0     seq
    ............................................................ccugcugggguggaaccuggucu.......................    3        0     seq
    .............................................................cugcugggguggaaccugguc........................    4        0     seq
    .............................................................cugcugggguggaaccuggucu.......................    3        0     seq
    ..............................................................ugcugggguggaaccuggucugu.....................    18       0     seq
    ...............................................................gcugggguggaaccuggucugu.....................    2        0     seq
```
